# The effects of different stress on intestinal mucosal barrier and intestinal microecology were discussed based on three typical animal models

**DOI:** 10.3389/fcimb.2022.953474

**Published:** 2022-09-29

**Authors:** Junfeng Guo, Xiaokun Lou, Wenyan Gong, Jing Bian, Yuhan Liao, Qi Wu, Qibin Jiao, Xingwei Zhang

**Affiliations:** ^1^ Department of Clinical Medicine, Affiliated Hospital of Hangzhou Normal University, Hangzhou Normal University, Hangzhou, China; ^2^ Department of Cardiology, Affiliated Hospital of Hangzhou Normal University, Hangzhou, China

**Keywords:** stress, burns, intestinal ischemia-reperfusion injury, depression, intestinal microecology, intestinal mucosal barrier

## Abstract

Recent studies have revealed that the effect of intestinal microecological disorders on organismal physiology is not limited to the digestive system, which provides new perspectives for microecological studies and new ideas for clinical diagnosis and prevention of microecology-related diseases. Stress triggers impairment of intestinal mucosal barrier function, which could be duplicated by animal models. In this paper, pathological animal models with high prevalence and typical stressors—corresponding to three major stressors of external environmental factors, internal environmental factors, and social psychological factors, respectively exemplified by burns, intestinal ischemia-reperfusion injury (IIRI), and depression models—were selected. We summarized the construction and evaluation of these typical animal models and the effects of stress on the organism and intestinal barrier, as well as systematically discussed the effects of different stresses on the intestinal mucosal barrier and intestinal microecology.

## Introduction

Stress refers to the adaptive changes and reconstruction of the homeostasis of the internal environment in order to meet needs when the body is strongly stimulated by various factors (Charmandari et al., 2005). But, when this stimulus exceeds the degree of the organism’s ability to compensate, it leads to a series of pathological changes in the organism. Factors that trigger the stress response of the organism are collectively called stressors, which could be divided into three major categories according to their sources: external environmental factors, internal environmental factors, and social psychological factors.

Recent study has found that chronic stress promoted increased systolic blood pressure, increased urinary protein, and glomerular damage in 5/6 nephrectomy injury mice, which could be prevented by cathepsin K deficiency or pharmacological inhibition. Chronic stress is also a risk factor for cardiovascular disease (Yue et al., 2022). Besides, cathepsin S plays an important role in stress-related chronic intimal hyperplasia and chronic stress accelerates the degradation of GLP-1 mediated by Dipeptidyl Peptidase-4(DPP4) (Piao et al., 2017; Wang et al., 2019). These results are a new therapeutic target for the control of vascular aging, vascular remodeling, and restenosis associated with chronic psychological stress. Moreover, a study on hematopoietic stem cell activation in response to chronic stress suggested that inhibition of DPP4 or stimulation of GLP-1R may have application value in the treatment of inflammatory diseases (Zhu et al., 2017). Thus, the impact of stress is extensive, so it is of great significance to explore its specific mechanism to prevent emergency damage.

Stress also causes damage to the intestinal mucosal barrier, which further leads to intestinal microecological disorders. Under normal circumstances, the intestine relies on the mucosal barrier consisting of a mechanical barrier, biological barrier, immune barrier, and chemical barrier to selectively transport relevant substances, and at the same time effectively prevent bacteria and endotoxins in the intestinal cavity from passing through the intestinal mucosal barrier (Chen et al., 2021), thus maintaining the health of intestine and body. However, an organism prioritizes the blood and energy supply to vital organs when it suffers severe or prolonged situations. The redistribution of blood may result in insufficient effective blood circulation in the intestine and reduce the energy supply of intestinal epithelial cells, leading to a series of pathophysiological changes such as reduced bile secretion or enterohepatic circulation disorder. These alterations disrupt the intestinal barrier and further cause intestinal dysfunction. In addition, intestinal mucosa injury also triggers bacterial translocation (BT), systemic bacterial infection, or even systemic organ dysfunction and circulatory failure, which endanger patients’ lives. Therefore, prevention and controlling the damage of intestinal mucosal barrier function under various pathological stresses are important parts of the prevention and treatment of enteric-derived infections today, and it is of significance to understand the effects of stressors on the intestine and their pathophysiological basis to achieve good prevention and treatment effects.

## Subsections relevant to the subject

### 1 Typical animal models and evaluation criteria of different stressors

#### 1.1 Exogenous stress-extensive burns

##### 1.1.1 Model establishment

The burn model was established according to the principle of flame burn, in general burning for 15 s causes deep-partial thickness burns (deep 2^nd^ degree). After being anesthetized and secured, the mice were shaved of the hair on the pre-modeling area of the back using 8% sodium sulfide solution. A piece of paper, controlling the burn area to about 10% of the body surface, was placed on the back of the mice and ignited by adding a drop of 95% ethanol, and extinguished with a wet cloth after burning for 15 s. 0.3-0.4 mL glucose saline was injected intraperitoneally immediately after the burning, and 0.2-0.3 mL milk by gavage on the second day. Data collection could be started on the third day. This method is simple and easy to perform without additional instruments and the control group should be treated with a pseudo-burn, i.e. shaving and hair removal after anesthesia only, which was reported by the study of Abdullahi A (Abdullahi et al., 2014). Considering that the anesthetized mice still had symptoms such as back skin folds and even general convulsions and were easily burned directly by 8% sodium sulfide aqueous solution, pre-experiments should be conducted before the formal experiment to determine the burn time, burn level, and whether to shave to achieve the best results.

##### 1.1.2 Model evaluation

The evaluation of burn grade is mainly based on the histopathological changes of burned skin. The keratinocytes, clear skin appendages, and dermal collagen fibers were observed in normal mice. Superficial thickness (1^st^ degree) and superficial-partial thickness (2^nd^ degree) damage the superficial skin layer and superficial dermis, deep 2^nd^ degree triggers the deep dermis layer and still left skin attachments, and full thickness (3^rd^ degree) induces the subcutaneous, fat, muscle and bone injury, and so on (Morarasu et al., 2021). Repair of the burn wound, healing, prevention, and treatment mainly focuses on deep 2^nd^ degree burns, which harm the deep dermis, inducing unclear keratinocyte structure, obvious necrosis, partial cells lysis, collagen fiber breakage, and skin appendages such as sebaceous glands and hair follicles cannot be clearly observed, while the muscle cell structure is intact.

The evaluation of burn grade should not be delayed too long and HE staining of burned skin should be performed on the 3^rd^ day after burning for evaluation, considering the newborn hair follicles will appear in the deep dermis 2 to 3 days after skin trauma in mice (Takeo et al., 2015).

#### 1.2 Endogenous stress-intestinal ischemia-reperfusion injury

##### 1.2.1 Model establishment

Mice should be fasted for 24 h before the establishment of IIRI models. After being anesthetized and secured, mice were shaved with the hair on the surgical site of the abdomen. A median incision was made under the scabbard to expose the right abdominal cavity and superior mesenteric artery (SAM), and clamp the SAM root with a non-invasive micro-arterial clip. When the disappearance of SAM pulsation, swelling of the intestine and gradual whitening of the intestinal wall appears, indicating the blood flow is successfully blocked. Isotonic sodium chloride solution was intermittently injected into the abdominal cavity or onto the gauze covering the abdominal cavity during clamping to prevent and alleviate the transient blood volume reaction, which occurred after loosening the arterial clip (Zhu et al., 2006). Release the arterial clip after a period of ischemia, and the color of the intestinal tube changed to pink, suggesting blood flow restoration and ischemia-reperfusion happened. Putting the intestinal tissue back *in situ* as much as possible and suturing the abdomen to avoid intestinal obstruction and reduce mortality. Ischemia time can be determined by 45 min, 60 min, 75 min, and 90 min after pre-experimentation and injury scoring.

##### 1.2.2 Model evaluation

The evaluation of IIRI includes abnormal behaviors of mice, histopathological sections of ischemic intestinal segments, and intestinal water content.

- Behavioral changes. After awakening from anesthesia, IIRI mice showed obvious painful postures such as head down and back arching, responsiveness decreases, and activities reduce.
- Intestinal histopathological changes. Intestinal mucosa ulcer and hemorrhage, intestinal structure disorder, villus structure fracture, fall off or basic disappearence, intestinal gland reduction, capillary hyperemia and edema, and inflammatory cells infiltration can be observed in histopathological sections of ischemia-reperfusion injury sites. The specific evaluation is based on Chiu’s scoring criteria for IIRI model as reported by Chiu (Chiu et al., 1970).
- Increased intestinal water content. A part of the IIRI segment was taken to weigh the intestine wet weight (W1) and the dry weight (W2) after constant temperature baking to completely remove the water with an analytical balance. The intestinal water content (%) = [(W1-W2)/W1] × 100%.

#### 1.3 Psychosocial stress-chronic social defeat stress

Psychological stress generated by psychosocial stressors such as emotion, family, growth experience, living environment, and disasters is an important cause of depression. Researchers have constructed various stress-induced depression animal models in the past, but psychological stress is caused by artificially imposed environmental stress, all of which have social properties. Therefore, in addition to its characteristics of persistent behavioral and biological changes, CSDS can simulate the pathological process of chronic psychosocial stress and progressive development of depression to a greater extent, making it one of the best models to explore chronic psychological stress and depression.

##### 1.3.1 Model establishment

The specific construction methods refer to the study by Golden S A (Golden et al., 2011). The key to the establishment of the CSDS model is making a mouse be continuously attacked by “aggressive mice”, and the attacked mouse suffered persistent maladjustment to the attack due to an inability to seek help and environmental stress, which means the persistent social defeat triggers the development of psychological stress. Ten days later, the attacked mice showed crouching, bowing, reduced behavior, and even giving up attempts to escape, suggesting depression-like behavior occurs and the model evaluation can be further proceeded (Berton et al., 2006).

##### 1.3.2 Model evaluation

The evaluation of depression-like behavior analysis includes sucrose preference test, sociability test, forced swimming test, tail suspension test, novel object recognition, elevated plus maze, Morris water maze, Y maze, and so on (Bevins and Besheer, 2006; Walf and Frye, 2007; Hölter et al., 2015; Kapadia et al., 2016). The sucrose preference test detects the depression pleasure deficit degree. Novel object recognition evaluates the memory ability for familiar objects. Morris water maze evaluates the spatial memory ability and learning ability. Elevated plus maze evaluates anxiety-like behavior. The sociability test directly reflectes the social avoidance behavior of depressed mice. The rest were classical experiments to evaluate the depression-like behavior of mice. This paper introduces four classic tests with simple apparatus and convenient operation.

- Sucrose preference test (Willner et al., 1987). A greater rate of sucrose water preference indicates a more severe depressive pleasure deficit in the experimental subject. This experiment is simple and only needs normal ultra-pure water at high pressure and 1% sucrose water. Two bottles of water were placed in a suitable area for the subject to drink freely and the two bottles are swaped every 12 h to avoid the subject’s position preference. The two bottles of water were withdrawn and fasted for 24 h after four exchanges. Following, the two bottles were placed randomly for 1 h, and calculated the 1% sucrose water consumption (A) and ultra-pure water consumption (B). The sucrose preference index (%) = A/(A + B) × 100%.
- Sociability test (Xu et al., 2019). Behavioral tests were performed during the daytime and the experimental devices consist of a video analysis system, a rectangular box, and two square metal cages that can accommodate just one mouse, being placed diagonally on both sides of the rectangular box. Firstly, the subjects were acclimatized in the test chamber for 30 min before recording. Secondly, the subjects were placed inside the rectangular box for 5 min to adapt to unfamiliar mice in the upper right corner of the metal cage. Thirdly, the subjects were allowed to move freely in the rectangular box for 10 min with an unfamiliar female mouse of the same age in the upper right metal cage, and record the data of path trajectories, number of interactions with female mice, and time spent in diagonal areas, etc.
- Forced swimming test. Add about 2L of water of 22 ± 1°C in a 3L beaker to ensure the water is deep enough to prevent the subject standing upright and force them to float. Mice will try to escape when forced to swim, but the inescapable oppressive environment makes them show a typical “immobile state”. The subject was placed in the beaker for 15 min on the first day and for 6 min on the next day, and the immobile time for the latter 4 min should be recorded.
- Tail suspension test. Similar to the forced swimming test, it is a classic experiment to evaluate depression-like behavior in animals (Yan et al., 2014). The mouse is secured by the tail by a rubber band, suspended about 30-40 cm from the ground, and its behavior recorded for 6 min and the immobile time for the latter 4 min.

## 2. Overview of different stressors and effects on intestinal microecology

### 2.1 Burns

Burns are a common type of accidental injury caused by flame, hot liquid, steam, electricity, incandescent metals, etc. (Smolle et al., 2017), which are classified as 1^st^ degree, 2^nd^ degree, deep 2^nd^ degree, and 3^rd^ degree according to the severity of the burn trauma. Skin microcirculation changes occur locally only in the acute period of 3-4 days after light burns, while in serious cases, distant organs like the intestine will show microcirculation adjustment disorders. When burned, the patient’s skin is bare, and the mucous membrane is damaged, which directly destroys the natural barrier between the organism and the outside, and the bacteria and toxins in the external environment can easily infect it. Extensive burns cause protein loss, and lead to immune system function injury, while massive use of antibiotics will cause gastrointestinal dysfunction, and further trigger internal and external infection and aggravation of the condition. BT occurs in severe burns patients, which leads to the arrival of bacteria through the mesenteric vein system to the portal vein and intestinal lymphatic system to systemic circulation (Ciftci et al., 2012), further endangering patients’ lives due to systemic inflammatory response syndrome (SIRS) or multiple organ dysfunction syndrome (MODS).

In the early stage of burns, there is a transient constriction of blood vessels in the burn area, the blood flow in the capillaries slows down, and leukocytes attach to the capillaries and small veins causing poor blood flow and promoting thrombosis. In addition, blood viscosity increases due to the aggregation of red blood cells and platelets, and the slowing down of the flow speed and forms vortices, which further promotes the formation of thrombosis (Liu et al., 2021). Microvessels dilate reduces vascular resistance and promotes blood flow, but excessive dilatation will cause blood pressure to decrease and blood flow blocked, leading to bruising of tissues and organs (Jeschke et al., 2020). Besides, microvessels dilate also increases the permeability of some microvenules and capillaries, as well as leads to the leakage of some blood components, and further triggers edema and decrease in plasma colloid osmotic pressure, which are the primary causes of body fluid loss or even shock in the early stages of the burn (Hu et al., 2015). Due to neutrophil aggregation, massive release of inflammatory mediators such as pro-inflammatory cytokines and oxygen radicals, and activation of related transduction pathways (Agay et al., 2008; Cevik et al., 2012; Kim et al., 2012; Chi et al., 2021), the inflammatory response damages the vascular endothelium, changes in endothelial cell morphology, and increases microvascular permeability, which triggers massive leakage of albumin into the tissue space and causes secondary changes such as systemic edema, thoracic and abdominal exudate (Rizzo et al., 2016). The decreased effective circulating blood volume further develops into multiple organ failure (MOF), shock, and even death.

Ischemia and hypoxia of the gastrointestinal tract are the main manifestations of the intestinal effects of burns. Burn injury leads to a large loss of body fluids. For ensuring the blood supply to the heart, brain, and other vital organs, the sympathetic-adrenomedullary system of the body is excited to redistribute blood in the body, causing in the gastrointestinal tract a state of ischemia and hypoxia (He et al., 2019), which leads to structural damage to the intestinal mucosa, increased permeability, cell necrosis, apoptosis, and ulcer formation.

Intestinal microorganisms play crucial roles in maintaining the integrity of the intestinal mucosal barrier and intestinal microecological balance. Burned mice mainly exhibited a reduced abundance of Bacteroidetes and an increased abundance of Phylum Firmicutes. When severe burn happened, the genera Mollicutes_RF9_g_norank, Candidatus_Saccharimonas, Butyricimonas, Rumino- coccaceae_UCG_010, and Ruminiclostridium_6 were increased (Feng et al., 2019). It found that the reduction of Clostridium butyricum and butyric acid, which contribute to the maintenance of endostasis in the intestinal flora, was particularly significant and negatively affected the intestinal mucosal barrier (Zhang et al., 2020). Oral administration of Clostridium butyricum to mice increased butyric acid levels, decreased TNF-a and IL-6 levels, and inhibited intestinal injury in burned mice. Burn-induced intestinal mucosal barrier damage and intestinal microecological disorders may be a potential mechanism for burn-induced sepsis (Beckmann et al., 2018). Intestinal bacteria and bacterial endotoxins invade through the damaged intestinal mucosa epithelium and reach mesenteric lymph nodes, causing bacterial translocation and imbalance of the intestinal flora ratio and intestinal micro-ecosystem, and further producing masses of potentially pathogenic substances and promoting systemic infection (Wang et al., 2019).

Considering that the traditional debridement treatment caused secondary injury to the wound, so the modified moist exposure therapy with moist burn cream was used. Under treatment, it should not ignore the protection of the intestinal tract, and it is vital to monitor intestinal function in time to avoid intestinal microecological disorder and BT.

### 2.2 IIRI

The pathogenesis of exogenous stress exhibits microcirculatory disorders and intestinal ischemia. The intestinal tract is highly sensitive to ischemia-reperfusion injury, which is the pathological processes of blood flow restoration and follow-up response after ischemia. Considering that transient ischemia leads to substantial damage to local mucosa and there is no uniform clinical treatment for IIRI, it is necessary to discuss the specificity of IIRI as endogenous stress.

Apoptosis, calcium overload, oxidative stress, and inflammatory and immune responses are associated with IIRI. Increased immune cell and mast cell activation promotes the release of inflammatory mediators, causing an inflammatory response and aggravating the injury. The conversion of xanthine dehydrogenase to xanthine oxidase in intestinal epithelial cells generates excessive oxygen free radicals, which directly or indirectly damage the DNA, proteins, and enzymes (Bhattacharyya et al., 2014). Increased anaerobic metabolism and free radicals after intestinal mucosal injury causes impaired calcium pump and induces calcium overload, which leads to the increase in intracellular calcium ion concentration and further trigger mitochondrial dysfunction and related enzyme activation, eventually causing apoptosis.

Intestinal microbes play a role in maintaining intestinal immune function and the intestinal mucosal barrier. It has been shown that when broad-spectrum antibiotics are taken it disrupts the normal colonization of the intestine, which weakens the intestinal barrier and immune function, and further exacerbates the injury in intestinal ischemia-reperfusion (Chen et al., 2008). After IIRI, microorganisms in the intestine leave the usual site of residence and penetrate from the injury into the intestinal mucosa and submucosa (Nadatani et al., 2018), and also through the intestinal mucosal microcirculation into other organs, thus inducing inflammation.

After IIRI, the composition of colonic bacteria in mice was significantly disturbed. The relative abundance of Bacteroidetes and Phylum Firmicutes was significantly increased, while the relative abundance of Mycobacterium verrucosum was decreased (Deng et al., 2022). Metabolomic results showed that the biosynthesis of secondary metabolites and polysaccharides and the genomic abundance of metabolic pathways were significantly impaired after IIRI, and the capsiate in microbial metabolites decreased (Deng et al., 2021). Besides, some strains have been found to exacerbate injury. The presence of P. aeruginosa in the distal intestine may enhance the lethal effects of IIRI (Fink et al., 2011), and single colonization of Escherichia coli strain JP313 enhances leukocyte adhesion to small mesenteric veins with IIRI (Ascher et al., 2020).

At present, more and more enteric bacteria have been found to have the potential to treat intestinal and extraintestinal organ damage caused by intestinal IIRI. But since the pathogenesis of IIRI is not yet fully understood, clinical treatment or prevention is mostly aimed at alleviating acute injury by simulating several links in the process of IIRI, such as ischemic preconditioning and ischemic postconditioning (Wong et al., 2021; Mi et al., 2021).

### 2.3 Depression

The morbidity of depression is increasing and the suicide rate of depression groups is higher than those without, accompanying the increasing living standards and material abundance, as well as the spiritual dissatisfaction and challenges (Cavanagh et al., 2003). Psychosocial problems are a major risk factor for depression. Except for psychological problems, some physiological changes will appear when depression is in an advanced stage. Studies demonstrated that depression is associated with decreased levels of 5-hydroxytryptamine (5-HT) and norepinephrine, for which some targeted drugs, like isoniacyl isprehydrazine and promimazine, have been used in clinical treatment according to monoamine theory.

Depression is profoundly associated with some intestinal disorders, acts in both directions with intestinal diseases, and has relevance in their pathogenesis. The reports of psychosomatic factors on the pathogenesis of ulcerative colitis (UC) indicate that negative psychological stimuli affect the neuro-endocrine immune system and brain-gut axis system by altering the secretion of transmitters like 5-HT, leading to abnormal secretion of immune factors such as IL-2, IL-6, and IL-8 (Feuerstein et al., 2019; Peppas et al., 2021). These immune factors have a certain pro-inflammatory effect on the pathogenesis of UC, and promote the secretion of 5-HT feedback through the neuro-endocrine, immune, and brain-gut systems, aggravating the disease and further worsening the intestinal inflammation.

Studies showed that Phylum Firmicutes significantly reduced in depressed patients (Huang et al., 2018). Besides, the Phylum Firmicutes decreased and Bacteroidetes increased in Major Depressive Disorder (MDD) patients, which lead to a reduced ability to produce short-chain fatty acids (SCFA) (Liu et al., 2020). Phylum Firmicutes contribute to the fermentation of carbohydrates into SCFA, and SCFA deficiency may weaken the function of the intestinal barrier, leading to the crossing of pathogens and metabolites, which induce an immune response that may be associated with the onset and development of depression (Duncan et al., 2007).

## 3. Effects of different stressors on intestinal mucosa

### 3.1 Reasons for burn-induced intestinal mucosal injury

Burns are a common cause of intestinal mucosal injury. Studies showed that burns increase intestinal permeability and tissue damage, allowing bacteria to enter the bloodstream and causing damage to the organism (Costantini et al., 2009a). The tight junction (TJ) between intestinal epithelial cells plays a crucial protective role in preventing bacteria. When burned, inflammation promotes the destruction of the tight connection structure mainly through the myosin light chain kinase (MLCK) signaling pathway.

#### 3.1.1 Disruption of tight junctions induced intestinal mucosal damage

TJs are composed of proteins located on the cell membrane and maintains the integrity of the intestinal mucosa, such as claudin, occludin, and the ZO family, and the absence of these proteins will damage the intestinal epithelial barrier (Shen et al., 2011). Changes in TJs after severe burns are mainly twofold. On the one hand, TJs in intestinal epithelial cells redistribute. Chuanli Chen found that when mice underwent 30% burns for 6 h, intestinal wall permeability changed and claudin-1, occludin and zonula occludens-1 (ZO-1) redistributed (Chen et al., 2012). Besides, Costantini also found the rearrangement of occludin and ZO-1 (Costantini et al., 2009b). On the other hand, changes were found in the expression of TJ. Reduced levels of occludin and ZO-1 expression were also found in Costantini’s study, and remained lower even after 24 hours than in the control group, suggesting that burn injury has an important effect on the expression of TJ.

#### 3.1.2 Molecular mechanism of TJ disruption

**Figure 1:**
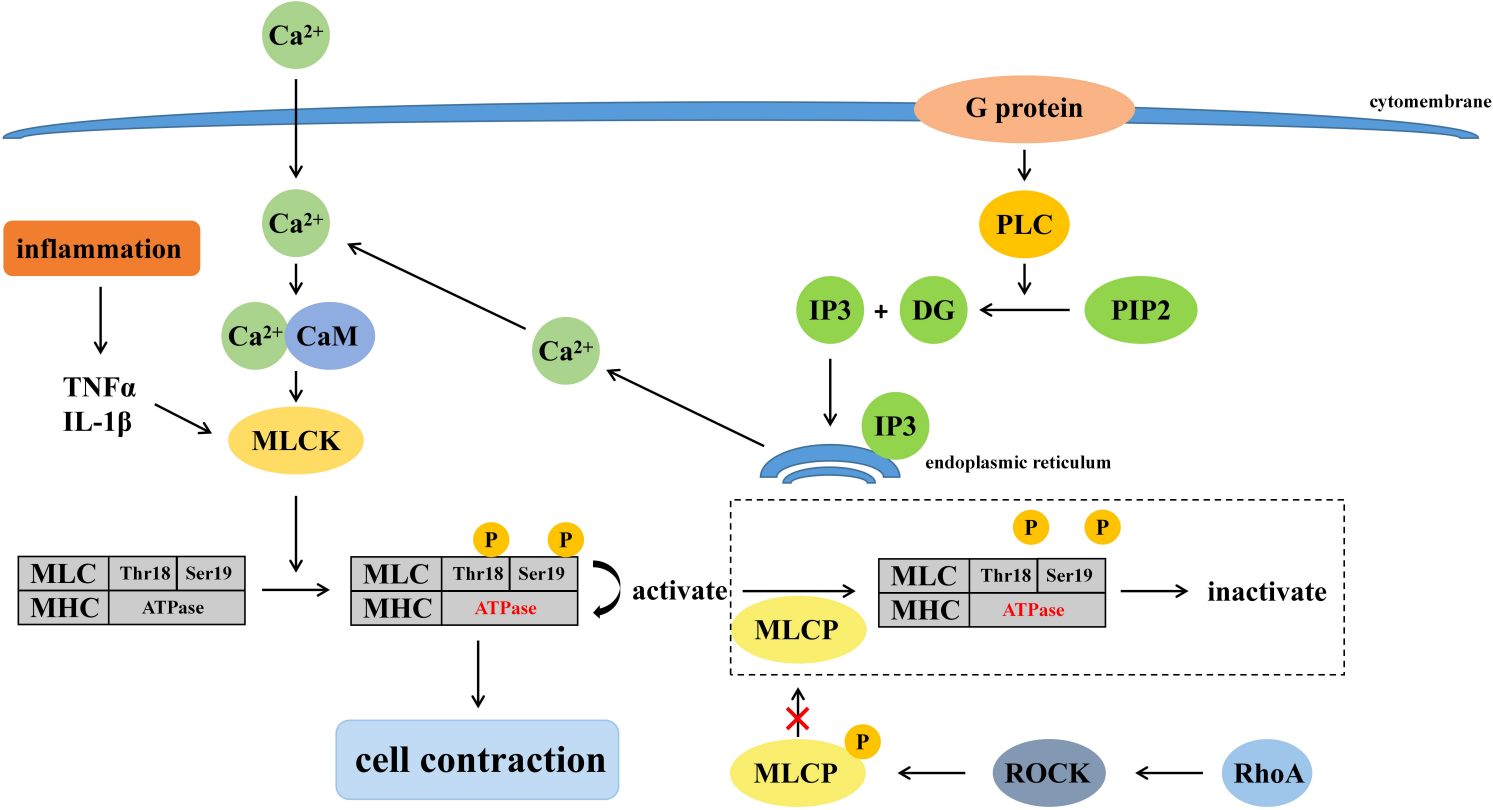
Mechanisms of cell contraction induced by burns.

The permeability of the intestinal mucosal barrier is related to the regulatory mechanisms of the cytoskeleton since TJs are connected to the cytoskeleton through ZO proteins (Fasano, 2008). Many signaling pathways involved in the cytoskeleton regulate intestinal mucosal barrier, such as phospholipase C-dependent signaling pathway, Ca2+-E cadherin signaling pathway, tyrosine kinase-phosphatase signaling pathway, Rho-Gtase pathway, MLCK signaling pathway, etc. MLCK signaling pathway plays a major role in the regulation of tight junctions, while phospholipase C-dependent signaling pathway and Rho-GTPase pathway can regulate MLCK signaling pathway. Mechanisms of cell contraction induced by burns are presented in **Figure 1**.

##### 3.1.2.1 Relationship between MLCK signaling pathway and TJ

It has been shown that MLCK regulates TJ (Cunningham and Turner, 2012). When MLCK is activated by calmodulin, etc., phosphorylation of residues at Thr18 and Ser19 of the myosin light chain (MLC) increased. Phosphorylated-MLC activates the ATPase of the myosin heavy chain, causing shrinkage of downstream cells, shortening of gaps between cells, and disruption of TJs (Zahs et al., 2012). The expression of MLCK was also associated with mucosal damage in addition to its activation. Multiple types of MLCK were generated due to the differences in MLCK promoters, among which smooth muscle myosin light chain kinase (smMLCK) and non-muscle myosin light chain kinase (nmMLCK) participate in the regulation of cell contraction *via* myosin. When activated by Ca2+, smMLCK induced cell contraction while nmMLCK did not (Lazar and Garcia, 1999). However, under inflammation, like burns, the transcriptional levels of smMLCK will be enhanced corresponding to the increase of MLCK, which destroys the TJ and further enlarges the intercellular space (Ye and Ma, 2008). In addition, burns prompt a massive release of cytokines and other inflammatory mediators such as TNF-α and IL-1β, which compete for herpesvirus receptors on T cells, upregulate MLCK transcription and exacerbate the destruction of the intestinal epithelial mucosa, further causing multifunctional organ failure, sepsis, even life-threatening conditions (Al-Sadi and Ma, 2007; Feng et al., 2019).

##### 3.1.2.2 Relationship between phospholipase C-dependent signaling pathway and MLCK signaling pathway

Burns activate G proteins mediated phospholipase C (PLC), which breaks down phosphatidylinositol (Piao et al., 2017; Zhu et al., 2017) bisphosphate (PIP2) into inositol triphosphate (IP3) and diacylglycerol (DG). IP3 binds to its receptors on the endoplasmic reticulum to release Ca2+, which activates the MLCK signaling pathway and promotes phosphorylation of MLC, ultimately leading to the disruption of TJ (De Bock et al., 2013).

##### 3.1.2.3 Relationship between Rho-GTPase pathway and MLCK signaling pathway

The Ras homologous (Rho), a member of the Ras superfamily of GTPases, mainly plays a regulatory role between actin and myosin in the intestinal mucosal epithelial barrier, and its mechanism of affecting TJ is also related to MLCK. Rho GTPases are divided into Rho (RhoA, RhoB, and RhoC), Rac (Rac1, Rac2, and Rac3), Cdc42 (Cdc42, RhoJ, and RhoQ), and RhoF (RhoD and RhoF) (El Masri and Delon, 2021). Rho-associated coiled-coil kinase (ROCK) is a kind of serine/threonine protein kinase (STPK), which is activated by RhoA. Myosin light chain phosphatase (MLCP) is phosphorylated and inactivated by activated-ROCK, and results in the inability to dephosphorylate MLC, further leading to an increase in phosphorylated-MLC and disruption of TJ (Xue et al., 2019). Studies have shown that intestinal mucosal reduced and permeability and phosphorylation of MLC decreased in burned mice injected with ROCK inhibitor (Liu et al., 2008). Therefore, the Rho-GTP pathway is also thought to be involved in the disruption of TJ in mice after burns.

### 3.2 Reasons of IIRI induced intestinal mucosal injury

**Figure 2:**
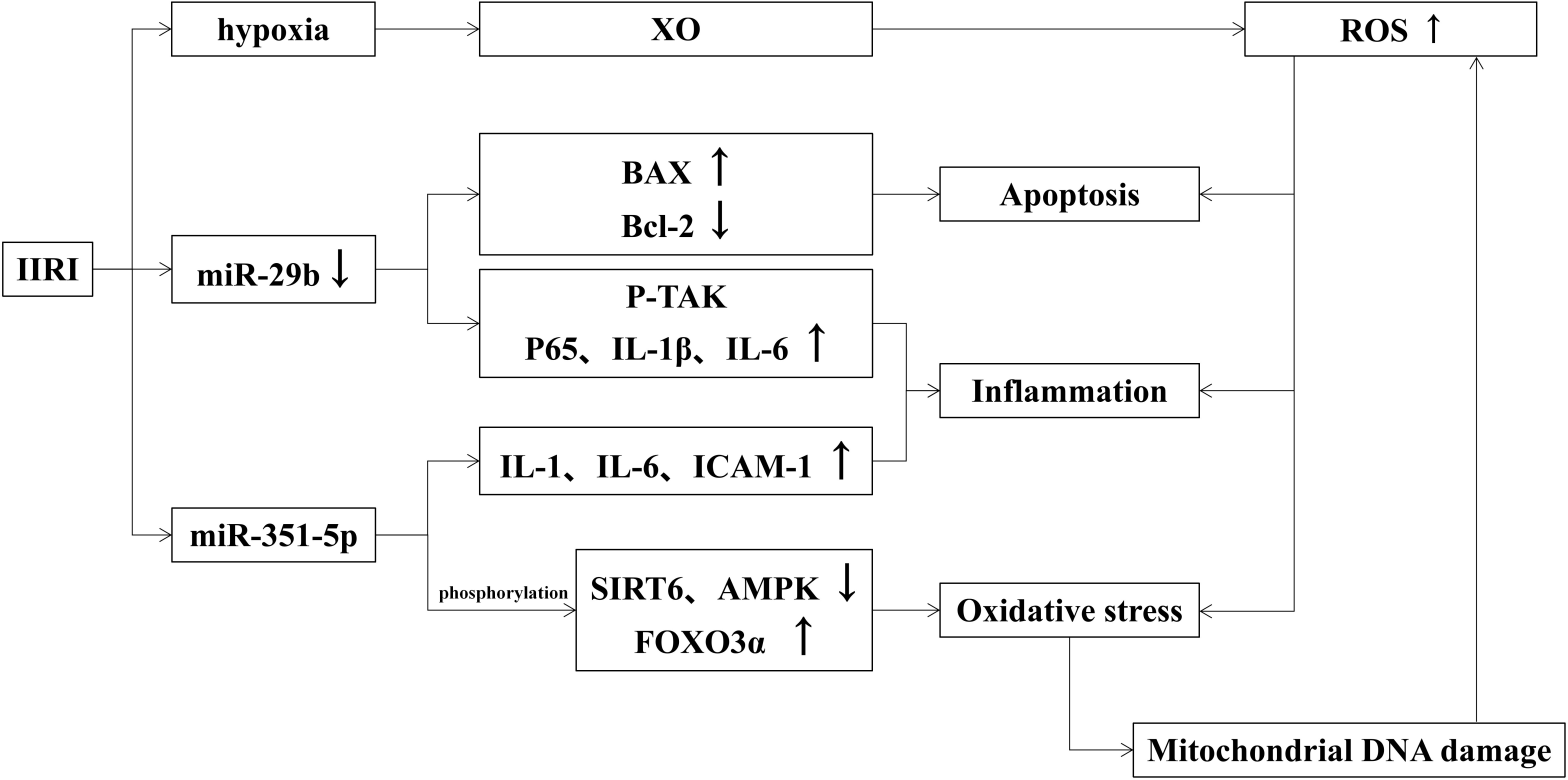
Effects of IIRI on intestinal mucosal injury.

When intestinal ischemia, the permeability of capillaries increases and causes fluid in the vessels to leak out into the tissue interstitial spaces, forming interstitial edema. If IIRI occurs, the capillary permeability of the intestinal wall will be further increased and even damage the intestinal mucosa. The effects of IIRI on intestinal mucosal injury are shown in **Figure 2**.

#### 3.2.1 Intestinal mucosal injury induced by oxidative stress in IIRI

It has been shown that a large number of reactive oxygen species (ROS) are generated during ischemia-reperfusion, which form lipid peroxides with the unsaturated fatty acids in the plasma membrane and activate the complement system to induce oxidative stress and further damage intestinal mucosal (Li et al., 2021). ROS are mainly derived from mitochondria in macrophages and neutrophils and produced to prevent intestinal pathogens from invading (Cieślar-Pobuda et al., 2017). The hypoxia-produced xanthine oxidase (XO) leads to ROS overload (Akinrinmade et al., 2016). Excess ROS also induces the production of inflammatory factors and apoptosis, which aggravate intestinal mucosa injury. The transfer of mitochondrial DNA also leads to increased oxidative stress (Yu and Bennett, 2016). Oxidative stress damages mitochondrial DNA and blocks the synthesis of the electron transport chain complex, leading to mitochondrial dysfunction. At the same time, the positive feedback of dysfunctional mitochondria increases the generation of ROS and exacerbates oxidative stress damage. Studies also found that oxidative damage exacerbates *via* the JAK/STAT signaling pathway (Wen et al., 2012), TLR receptor-mediated signaling pathway, and PKCβII/p66shc pathway in the organism (Fink et al., 2007; Wang et al., 2014). Similarly, Wen He demonstrated that ischemia-reperfusion could manifest as abnormal TJ between intestinal epithelial cells, enhancing intestinal permeability and causing focal hemorrhage and necrosis.

#### 3.2.2 The role of intestinal microorganisms in IIRI

Toll-like receptors (TLR) that recognize bacterial component molecules are closely related to intestinal mucosal injury, which plays a vital role in intestinal homeostasis and immune function in the intestine. It has been shown that the family member of TLR associated with IIRI is mainly TLR2 and TLR4, both of which can be induced to produce TNF-α and NF-κB by activation of relevant components of microorganisms. TLR2 is mainly activated by Gram-positive bacteria and peptidoglycan in fungi (Kawai and Akira, 2009), while TLR4 is mainly activated by lipopolysaccharide in Gram-negative bacteria (Hoshino et al., 1999). Some studies verify that TLR4 prevents increased intestinal permeability *via* TNF-α signaling to reduce IIRI, while others deem TLR4-mediated COX2 expression exacerbates intestinal mucosal injury (Moses et al., 2009; Zhu et al., 2017). Different effects produced by TLR4 may be related to various types of ligands.

#### 3.2.3 Roles of miRNAs in IIRI

A variety of miRNAs have been reported to play important roles in the maintenance of gastrointestinal homeostasis and immune function, as well as in the maintenance of intestinal mucosal barrier function (Akbari, 2020). MiR-29b, a member of the miR-29 family, is involved in the mediation of apoptosis, inflammatory response, and oxidative stress. When suffering IIRI, miR-29b expression is down-regulated which activates p-TAK signaling and promotes inflammatory response by up-regulating p65, IL-1β, and IL-6. Besides, decreased miR-29b induces apoptosis by up-regulating Bax and down-regulating Bcl-2. All above mentioned factors together exacerbate intestinal mucosal injury (Wang et al., 2017). Hu Y found that miR-351-5p induces oxidative stress by reducing SIRT6 and AMPK phosphorylation levels (Hu et al., 2018), as well as enhancing FOXO3α phosphorylation, IL-1, IL-6, and ICAM-1 expression. MiRNAs associated with IIRI reduce oxidative stress by decreasing SIRT1 and SIRT6, reduce apoptosis by inhibiting IGF-1/PI3K/Akt/mTOR pathway, Apaf-1, Cyt c, caspase-3, caspase-9, and PTEN, and reduce inflammatory response by inhibiting MAPKD1/NF-κB/IL-1, IL-6 and TNF-α inflammatory signaling pathways (Akbari, 2020).

### 3.3 Reasons of depression induced intestinal mucosal injury

Depression is a serious mental illness that negatively affects people’s thoughts and behaviors and poses a serious risk to human physical and mental health (De Zwart et al., 2019). Depression has been reported to disturb the homeostasis of the gastrointestinal tract and affects gastrointestinal tract function such as constipation, nausea, vomiting, etc (Bhatia and Tandon, 2005). Lina Wei found that chronic stress regulates the intestinal nervous system and secretory function to affect the activity of intestinal microorganisms, which counteract the intestinal mucosa causing intestinal mucosal damage and inflammation (Wei et al., 2019).

#### 3.3.1 Roles of microbiota-gut-brain axis in depression

The bidirectional link between brain and gut microbiota is known as the microbiota-gut-brain axis (Burokas et al., 2017), and signals from the brain affect the gut microbes through it. Conversely, microbes affect the central nervous system. According to Dabo Xu (Xu et al., 2014), chronic psychological stress causes changes in the number and type of intestinal communities, disrupting intestinal microecology (Ding et al., 2020). The disturbance of intestinal microorganisms produces lipopolysaccharides, increased inflammatory factors that damage the intestinal mucosa. Suzuki K found that psychological stress reduces α-defensin secreted by Paneth cells and destroys the diversity of intestinal microbes such as Firmicutes and Bacteroidetes (Suzuki et al., 2021).

#### 3.3.2 Roles of the immune-inflammatory pathway in depression

In patients with major depression, the immune-inflammatory pathway is overactivated, and the pro-inflammatory cytokines, T cell activation, and acute-phase protein increase and lead to intestinal inflammation (Sluzewska et al., 1996; Dowlati et al., 2010; Beurel et al., 2013). The increased levels of Oxidative and nitrative stress trigger the generation of ROS and reactive nitrogen species (RNS), resulting in impaired cell membrane structure and adhesion and further causing the destruction of intestinal wall cells (Maes et al., 2011).

#### 3.3.3 Roles of the hypothalamic-pituitary-adrenal axis in depression

Activation of the HPA axis promotes the release of catecholamine, and increases the activity of intestinal wall lymphocytes, macrophages, and neutrophils, which cause intestinal inflammation. Studies showed that chronic psychosocial stress increased the severity of 2, 4, 6-trinitrobenzenesulfonic acid (TNBS) and induced colitis in rats (Mawdsley and Rampton, 2005). Clinical studies also showed that chronic stress aggravated the course of enteritis, and the prognosis was easy to relapse (Gerbarg et al., 2015). Moreover, catecholamines enhanced IL-17A-induced neutrophil infiltration by activating the STAT3 signaling pathway in a model of enteritis induced by Dextran sulfate sodium (DSS) (Deng et al., 2016).

**Figure 3:**
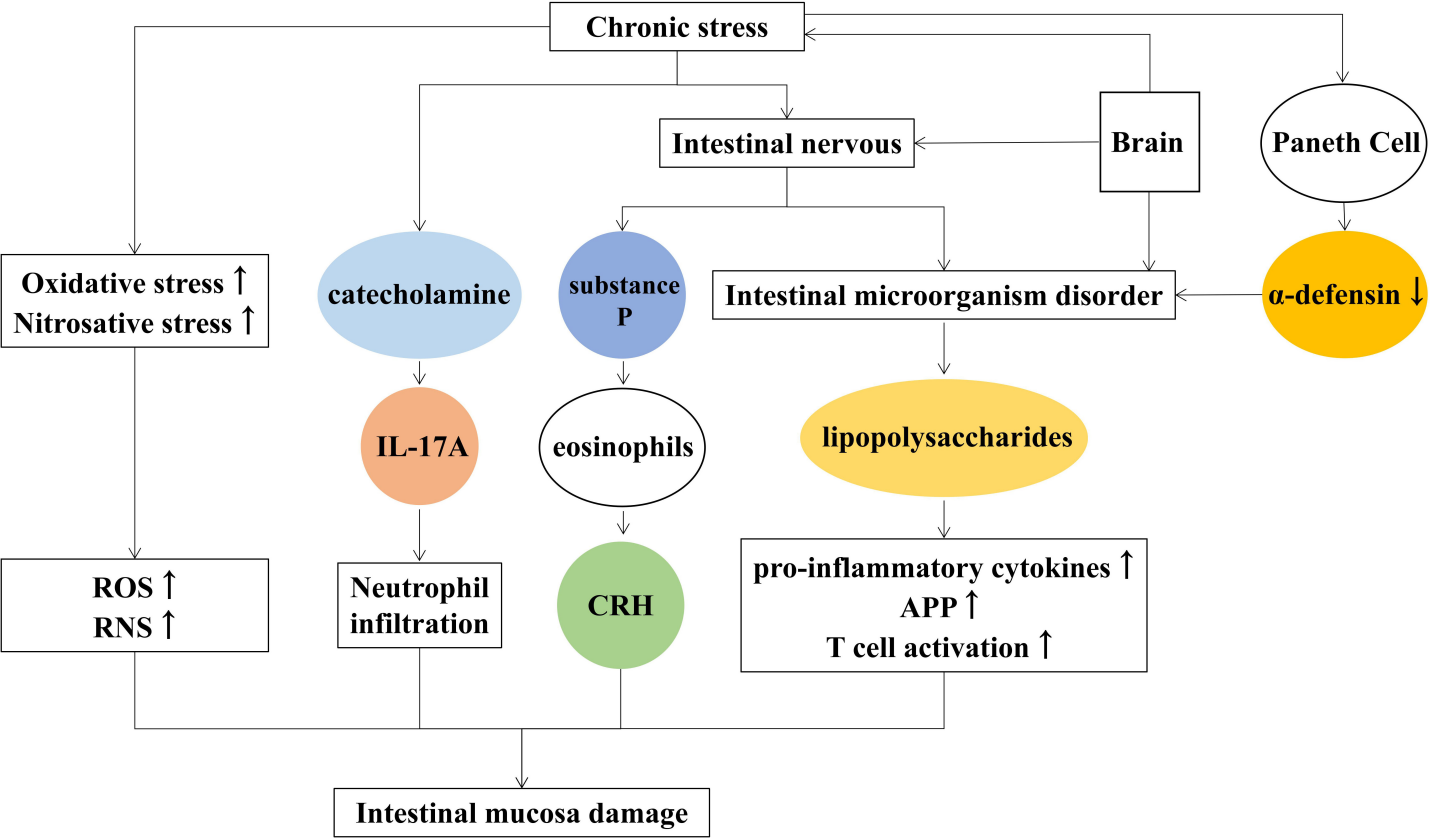
Mechanisms of chronic stress-induced intestinal mucosal injury.

In addition, chronic stress causes the release of substance P from intestinal nerve endings (Zheng et al., 2009), inducing the production of corticotropin-releasing hormone (CRH) by eosinophil and activating mast cells to induce intestinal epithelial barrier damage. The main reasons for chronic stress-induced intestinal mucosal injury are listed as follows (**Figure 3**).

## 4. Discussion

The growing evidence demonstrates that the intestinal mucosa is damaged under stress. The alteration of intestinal microecology and intestinal mucosal barrier induced by the above-mentioned three typical stressors is different (**Table 1**). Firstly, in terms of the time when the stress begins up to the appearance of the intestinal response, the shortest is IIRI, followed by burns, and the longest is depression (Karabeyoğlu et al., 2008; Cruz et al., 2011). Secondly, in terms of assays, the same detection of intestinal mucosal barrier damage induced by three typical stressors are TJ protein, fluorescein isothiocyanate (FITC)-dextran permeability, while the burn model also measures the MLCK activity, the ischemia-reperfusion model tests free radical content, and more attention was paid to the changes of gut microflora for the depression. Thirdly, in terms of intestinal microbiota changes, the abundance of intestinal Bacteroidetes decreased and Phylum Firmicutes increased when burned, Bacteroidetes and Phylum Firmicutes all increased in IIRI, and Bacteroidetes increased and Phylum Firmicutes decreased in depression. Fourthly, the differences between these three typical stresses on the intestinal response remain to be investigated because of the difference in the experimental parameters and the indicators detected. However, it is worth noting that there is also a temporary ischemia-reperfusion injury to the intestine in the burn model due to the large loss of body fluids and thus greater damage to the intestinal mucosa (Zhi et al., 2015).

**Table 1:** Difference between intestinal mucosal barrier injury and intestinal microecological imbalance caused by burning, IIRI, and depression.

		Burn	IIRI	Depression
Time		Medium	Shortest	Longest
Assays	Similarities	TJ protein, FITC-dextran, etc		
	Differences	MLCK	Free radical	microflora
microbiota	Bacteroideted	↓	↑	↑
	Phylum Firmicutes	↑	↑	↓

The symbols of ↓ means decrease and ↑ means increase.

## 5. Summary and outlook

The biological barrier consists of a stable proportion of intestinal flora that colonizes the surface of intestinal mucosa or resides in the intestinal lumen, which effectively prevents the invasion of harmful bacteria. The chemical barrier is composed of intestinal mucus and digestive juices secreted by intestinal epithelial cells, bacteriostatic substances produced by intestinal parasitic bacteria, and co-metabolites revised from them, which moderates intestinal peristalsis and permeability, and prevents the adhesion of toxins and pathogens. The structural and functional integrity of the intestinal mucosal epithelium and TJ between cells constitute a mechanical barrier that directly prevents bacterial endotoxins and bacteria from crossing. The secretory IgA (SIgA) and other antibodies secreted by submucosal lymphoid tissue, intestinal mucosal epithelial cells, and the interaction between immune molecules and immune cells constitute the immune barrier, which kills pathogens, maintains mucosal integrity, and its normal function. The structural integrity and functional coordination of the above four barriers together constitute the intestinal mucosal barrier, maintaining the microecological stability of the human intestine.

As a relatively common pathogenic factor, stress is prone to cause digestive and psychiatric disorders, which seriously affect the physical and mental health of patients. In recent years, more and more studies have shown that under stress, the intestinal mechanical barrier is damaged, and then the intestinal mucosal permeability increases, which brings a large amount of endotoxin into the blood and can even be life-threatening. The mechanical barrier is the most important layer of the intestinal mucosal barrier, which is composed of multiple proteins that work together to maintain the normal form and function of the intestinal mucosa. The imbalance of intestinal microecology, poor tissue perfusion, impaired blood circulation, prolonged lack of nutrition or ischemia and hypoxia lead to edema or atrophy of intestinal mucosal cells, enlarging the intercellular space and damaging the TJ. Once the TJ between epithelial cells is mutated or missing, the integrity of the mucosal barrier cannot be guaranteed. The toxic substances and bacteria in the intestinal cavity leak into the surrounding tissues through the intercellular space or enter the intestinal mucosal lymph nodes and portal vein system by cell bypass because of the increase of intercellular permeability, which activates the immune inflammation, destroys the intestinal epithelium again, further increase the permeability, aggravate the disease and even endangers the patient’s life. In this paper, we summarize the mechanisms of intestinal wall permeability increased by burns, intestinal ischemia-reperfusion injury, and depression. Inflammatory response and intestinal microecology disorders are the initial or aggravating factors of intestinal mucosal barrier damage and intestinal microecological disorder caused by these three stressors, and intestinal dysfunction further causes an inflammatory response. Selective inhibition of pathogenic bacteria in the intestinal tract and growth of conditioned pathogenic bacteria, reduction of drug-resistant strains, protection of intestinal mucosa, and maintenance of intestinal microecological balance are important means to prevent stress-induced intestinal dysfunction, which provide references for alleviating intestinal dysfunction or inducing endotoxemia in patients suffered stress. However, the mechanisms of stress-induced increased intestinal wall permeability are not completely clarified, and it still needs to be explored and solved.

## Author contributions

JG, XL, and WG drafted the manuscript. QW, JB, and YL helped to prepare the manuscript. WG, QJ, and XZ were involved in revising it critically. All authors contributed to the article and approved the submitted version.

## Funding

This work was supported by research grants from the National Natural Science Foundation of China (No. 81900745) and the Starting Foundation of Natural Science from Hangzhou Normal University (No. 2019QDL010).

## Conflict of interest

The authors declare that the research was conducted in the absence of any commercial or financial relationships that could be construed as a potential conflict of interest.

## Publisher’s note

All claims expressed in this article are solely those of the authors and do not necessarily represent those of their affiliated organizations, or those of the publisher, the editors and the reviewers. Any product that may be evaluated in this article, or claim that may be made by its manufacturer, is not guaranteed or endorsed by the publisher.

## References

[B1] Abdullahi A. Amini-Nik S. Jeschke M. G. (2014). Animal models in burn research. Cell Mol. Life Sci. 71 (17), 3241–3255. doi: 10.1007/s00018-014-1612-5 24714880 PMC4134422

[B2] Agay D. Andriollo-Sanchez M. R. Touvard L. Denis J. Roussel A. M. (2008). Interleukin-6, TNF-alpha and interleukin-1 beta levels in blood and tissue in severely burned rats. Eur. Cytokine Netw. 19 (1), 1–7. doi: 10.1684/ecn.2008.0113 18299267

[B3] Akbari G. (2020). Emerging roles of microRNAs in intestinal ischemia/reperfusion-induced injury: A review. J. Physiol. Biochem. 76 (4), 525–537. doi: 10.1007/s13105-020-00772-y 33140255

[B4] Akinrinmade F. J. Akinrinde A. S. Soyemi O. O. Oyagbemi A. A (2016). Antioxidant potential of the methanol extract of parquetina nigrescens mediates protection against intestinal ischemia-reperfusion injury in rats. J. Diet. Suppl. 13 (4), 420–432. doi: 10.3109/19390211.2015.1103828 26634775

[B5] Al-Sadi R. M. Ma T. Y. (2007). IL-1beta causes an increase in intestinal epithelial tight junction permeability. J. Immunol. 178 (7), 4641–4649. doi: 10.4049/jimmunol.178.7.4641 17372023 PMC3724221

[B6] Ascher S. Wilms E. Pontarollo G. Formes H. Bayer F. Müller M.. (2020). Gut microbiota restricts NETosis in acute mesenteric ischemia-reperfusion injury. Arterioscler. Thromb. Vasc. Biol. 40, 2279–2292. doi: 10.1161/ATVBAHA.120.314491 32611241 PMC7484055

[B7] Beckmann N. am P. Caldwell C. C. (2018). Burn injury alters the intestinal microbiome’s taxonomic composition and functional gene expression. PloS One 13, e0205307. doi: 10.1371/journal.pone.0205307 30289947 PMC6173435

[B8] Berton O. McClung C. A. Dileone R. J. Krishnan V. Renthal W. Russo S. J.. (2006). Essential role of BDNF in the mesolimbic dopamine pathway in social defeat stress. Science 311 (5762), 864–868. doi: 10.1126/science.1120972 16469931

[B9] Beurel E. Harrington L. E. Jope R. S. (2013). Inflammatory T helper 17 cells promote depression-like behavior in mice. Biol. Psychiatry 73 (7), 622–630. doi: 10.1016/j.biopsych.2012.09.021 23174342 PMC3582833

[B10] Bevins R. A. Besheer J. (2006). Object recognition in rats and mice: a one-trial non-matching-to-sample learning task to study ′recognition memory′. Nat. Protoc. 1 (3), 1306–1311. doi: 10.1038/nprot.2006.205 17406415

[B11] Bhatia V. Tandon R. K. (2005). Stress and the gastrointestinal tract. J. Gastroenterol. Hepatol. 20 (3), 332–339. doi: 10.1111/j.1440-1746.2004.03508.x 15740474

[B12] Bhattacharyya A. Chattopadhyay R. Mitra S. Crowe S. E. (2014). Oxidative stress: an essential factor in the pathogenesis of gastrointestinal mucosal diseases. Physiol. Rev. 94 (2), 329–354. doi: 10.1152/physrev.00040.2012 24692350 PMC4044300

[B13] Burokas A. Arboleya S. Moloney R. D. Peterson V. L. Murphy K. Clarke G.. (2017). Targeting the microbiota-Gut-Brain axis: Prebiotics have anxiolytic and antidepressant-like effects and reverse the impact of chronic stress in mice. Biol. Psychiatry 82 (7), 472–487. doi: 10.1016/j.biopsych.2016.12.031 28242013

[B14] Cavanagh J. T. Carson A. J. Sharpe M. Lawrie SM. (2003). Psychological autopsy studies of suicide: a systematic review. Psychol. Med. 33 (3), 395–405. doi: 10.1017/S0033291702006943 12701661

[B15] Cevik O. Oba R. Macit C. Cetinel S. Kaya O. T. C. Sener E.. (2012). Lycopene inhibits caspase-3 activity and reduces oxidative organ damage in a rat model of thermal injury. Burns 38 (6), 861–871. doi: 10.1016/j.burns.2012.01.006 22356815

[B16] Charmandari E. Tsigos C. Chrousos G. (2005). Endocrinology of the stress response. Annu. Rev. Physiol. 67, 259–284. doi: 10.1146/annurev.physiol.67.040403.120816 15709959

[B17] Chen L. W. Chang W. J. Chen P. H. Liu W. C. Hsu C. M. (2008). TLR ligand decreases mesenteric ischemia and reperfusion injury-induced gut damage through TNF-alpha signaling. Shock 30 (5), 563–570. doi: 10.1097/SHK.0b013e31816a3458 18317407

[B18] Chen C. Wang P. Su Q. Wang S. Wang F. (2012). Myosin light chain kinase mediates intestinal barrier disruption following burn injury. PloS One 7 (4), e34946. doi: 10.1371/journal.pone.0034946 22529961 PMC3329538

[B19] Chen S. Zhang C. He B. He R. Xu L. Zhang S. (2021). The role of lncRNAs in regulating the intestinal mucosal mechanical barrier. BioMed. Res. Int. 2021, 2294942. doi: 10.1155/2021/2294942 34820453 PMC8608538

[B20] Chi Y. Liu X. Chai J. (2021). A narrative review of changes in microvascular permeability after burn. Ann. Transl. Med. 9 (8), 719. doi: 10.21037/atm-21-1267 33987417 PMC8106041

[B21] Chiu C. J. Mcardle A. H. Brown R. (1970). Intestinal mucosal lesion in lowflowstates. Arch. Surg. 101 (4), 478–483. doi: 10.1001/archsurg.1970.01340280030009 5457245

[B22] Cieślar-Pobuda A. Yue J. Lee H. C. Skonieczna M. Wei Y. H. (2017). ROS and oxidative stress in stem cells. Oxid. Med. Cell Longev. 2017, 5047168. doi: 10.1155/2017/5047168 29018510 PMC5606094

[B23] Ciftci I. Ozdemir M. Aktan M. Aslan K. (2012). Bacterial translocation and intestinal injury in experimental necrotizing enterocolitis mode. Bratisl. Lek. Listy. 113 (4), 206–210. doi: 10.4149/bll_2012_047 22502749

[B24] Costantini T. W. Loomis W. H. Putnam J. G. Kroll L. Eliceiri B. P. Baird A.. (2009a). Pentoxifylline modulates intestinal tight junction signaling after burn injury: effects on myosin light chain kinase. J. Trauma 66 (1), 17–24. doi: 10.1097/TA.0b013e318191bb1f 19131801 PMC4251583

[B25] Costantini T. W. Loomis W. H. Putnam J. G. Drusinsky D. Deree J. Choi S.. (2009b). Burn-induced gut barrier injury is attenuated by phosphodiesterase inhibition: effects on tight junction structural proteins. Shock 31 (4), 416–422. doi: 10.1097/SHK.0b013e3181863080 18791495 PMC3445035

[B26] Cruz R. J. Jr Harada T. Sasatomi E. Fink M. P. (2011). Effects of ethyl pyruvate and other α-keto carboxylic acid derivatives in a rat model of multivisceral ischemia and reperfusion. J. Surg. Res. 165 (1), 151–157. doi: 10.1016/j.jss.2009.07.008 19959189

[B27] Cunningham K. E. Turner J. R. (2012). Myosin light chain kinase: pulling the strings of epithelial tight junction function. Ann. N. Y. Acad. Sci. 1258 (1), 34–42. doi: 10.1111/j.1749-6632.2012.06526.x 22731713 PMC3384706

[B28] De Bock M. Wang N. Decrock E. Bol M. Gadicherla A. K. Culot M.. (2013). Endothelial calcium dynamics, connexin channels and blood-brain barrier function. Prog. Neurobiol. 108, 1–20. doi: 10.1016/j.pneurobio.2013.06.001 23851106

[B29] Deng Q. Chen H. Liu Y. Xiao F. Guo L. Liu D.. (2016). Psychological stress promotes neutrophil infiltration in colon tissue through adrenergic signaling in DSS-induced colitis model. Brain Behav. Immun. 57, 243–254. doi: 10.1016/j.bbi.2016.04.017 27133786

[B30] Deng F. Lin Z. B. Sun Q. S. Min Y. Zhang Y. Chen Y. (2022). The role of intestinal microbiota and its metabolites in intestinal and extraintestinal organ injury induced by intestinal ischemia reperfusion injury. Int. J. Biol. Sci. 18 (10), 3981–3992. doi: 10.7150/ijbs.71491 35844797 PMC9274501

[B31] Deng F. Zhao B. C. Yang X. Lin Z. B. Sun Q. S. Wang Y. F.. (2021). The gut microbiota metabolite capsiate promotes Gpx4 expression by activating TRPV1 to inhibit intestinal ischemia reperfusion-induced ferroptosis. Gut. Microbes 13, 1–21. doi: 10.1080/19490976.2021.1902719 PMC8009132 33779497

[B32] De Zwart P. L. Jeronimus B. F. De Jonge P. (2019). Empirical evidence for definitions of episode, remission, recovery, relapse and recurrence in depression: A systematic review. Epidemiol. Psychiatr. Sci. 28, 544–562. doi: 10.1017/S2045796018000227 29769159 PMC7032752

[B33] Ding F. Wu J. Liu C. Bian Q. Qiu W. Ma Q.. (2020). Effect of xiaoyaosan on colon morphology and intestinal permeability in rats with chronic unpredictable mild stress. Front. Pharmacol. 11, 1069. doi: 10.3389/fphar.2020.01069 32765272 PMC7378849

[B34] Dowlati Y. Herrmann N. Swardfager W. Liu H. Sham L. Reim E. K.. (2010). A meta-analysis of cytokines in major depression. Biol. Psychiatry 67 (5), 446–457. doi: 10.1016/j.biopsych.2009.09.033 20015486

[B35] Duncan S. H. Louis P. Flint H. J. (2007). Cultivable bacterial diversity from the human colon. Lett. Appl. Microbiol. 44 (4), 343–350. doi: 10.1111/j.1472-765X.2007.02129.x 17397470

[B36] El Masri R. Delon J. (2021). RHO GTPases: from new partners to complex immune syndromes. Nat. Rev. Immunol. 21 (8), 499–513. doi: 10.1038/s41577-021-00500-7 33547421

[B37] Fasano A. (2008). Physiological, pathological, and therapeutic implications of zonulin-mediated intestinal barrier modulation: living life on the edge of the wall. Am. J. Pathol. 173 (5), 1243–1252. doi: 10.2353/ajpath.2008.080192 18832585 PMC2570116

[B38] Feng Y. Huang Y. Wang Y. Wang P. Wang F.. (2019). Severe burn injury alters intestinal microbiota composition and impairs intestinal barrier in mice. Burns. Trauma 7, 20. doi: 10.1186/s41038-019-0156-1 31312663 PMC6610819

[B39] Feuerstein J. D. Moss A. C. Farraye F. A. (2019). Ulcerative colitis. Mayo. Clin. Proc. 94 (7), 1357–1373. doi: 10.1016/j.mayocp.2019.01.018 31272578

[B40] Fink M. P. Macias C. A. Xiao J. Tyurina Y. Jiang J. Belikova N.. (2007). Hemigramicidin-TEMPO conjugates: novel mitochondria-targeted anti-oxidants. Biochem. Pharmacol. 74 (6), 801–809. doi: 10.1016/j.bcp.2007.05.019 17601494

[B41] Fink D. Romanowski K. Valuckaite V. Babrowski T. Kim M. Matthews J. B.. (2011). Pseudomonas aeruginosa potentiates the lethal effect of intestinal ischemia-reperfusion injury: the role of *in vivo* virulence activation. J. Trauma 71, 1575–1582. doi: 10.1097/TA.0b013e31821cb7e5 22002612 PMC3245766

[B42] Gerbarg P. L. Jacob V. E. Stevens L. Bosworth B. P. Chabouni F. DeFlilippis E. M.. (2015). The effect of breathing, movement, and meditation on psychological and physical symptoms and inflammatory biomarkers in inflammatory bowel disease: A randomized controlled trial. Inflammation Bowel. Dis. 21 (12), 2886–2896. doi: 10.1097/MIB.0000000000000568 26426148

[B43] Golden S. A. Covington H. E. Berton O. Russo S. J. (2011). A standardized protocol for repeated social defeat stress in mice. Nat. Protoc. 6 (8), 1183–1191. doi: 10.1038/nprot.2011.361 21799487 PMC3220278

[B44] He W. Wang Y. Wang P. Wang F. (2019). Intestinal barrier dysfunction in severe burn injury. Burns. Trauma 7, 24. doi: 10.1186/s41038-019-0162-3 31372365 PMC6659221

[B45] Hölter S. M. Garrett L. Einicke J. Sperling B. Dirscherl P. Zimprich A.. (2015). Assessing cognition in mice. Curr. Protoc. Mouse Biol. 5 (4), 331–358. doi: 10.1002/9780470942390.mo150068 26629775

[B46] Hoshino K. Takeuchi O. Kawai T. Sanjo H. Ogawa T. Takeda Y.. (1999). Cutting edge: Toll-like receptor 4 (TLR4)-deficient mice are hyporesponsive to lipopolysaccharide: evidence for TLR4 as the lps gene product. J. Immunol. 162 (7), 3749–3752. 10201887

[B47] Huang Y. Shi X. Li Z. Shen Y. Shi X. Wang L.. (2018). Possible association of firmicutes in the gut microbiota of patients with major depressive disorder. Neuropsychiatr. Dis. Treat 14, 3329–3337. doi: 10.2147/NDT.S188340 30584306 PMC6284853

[B48] Hu Y. Mao Z. Xu L. Yin L. Tao X. Tang Z.. (2018). Protective effect of dioscin against intestinal ischemia/reperfusion injury *via* adjusting miR-351-5p-mediated oxidative stress. Pharmacol. Res. 137, 56–63. doi: 10.1016/j.phrs.2018.09.016 30240824

[B49] Hu D. Yu Y. Wang C. Li D. Tai Y. Fang L. (2015). microRNA-98 mediated microvascular hyperpermeability during burn shock phase *via* inhibiting FIH-1. Eur. J. Med. Res. 20 (1), 51. doi: 10.1186/s40001-015-0141-5 25903459 PMC4411771

[B50] Jeschke M. G. van Baar M. E. Choudhry M. A. Chung K. K. Gibran N. S. Logsetty S.. (2020). Burn injury. Nat. Rev. Dis. Primers 6 (1), 11. doi: 10.1038/s41572-020-0145-5 32054846 PMC7224101

[B51] Kapadia M. Xu J. Sakic B. (2016). The water maze paradigm in experi-mental studies of chronic cognitive disorders: theory, protocols, analysis, and inference. Neurosci. Biobehav. Rev. 68, 195–217. doi: 10.1016/j.neubiorev.2016.05.016 27229758

[B52] Karabeyoğlu M. Unal B. Bozkurt B. Dolapçi I. Bilgihan A. Karabeyoğlu I.. (2008). The effect of ethyl pyruvate on oxidative stress in intestine and bacterial translocation after thermal injury. J. Surg. Res. 144 (1), 59–63. doi: 10.1016/j.jss.2007.02.050 17574580

[B53] Kawai T. Akira S. (2009). The roles of TLRs, RLRs and NLRs in pathogen recognition. Int. Immunol. 21 (4), 317–337. doi: 10.1093/intimm/dxp017 19246554 PMC2721684

[B54] Kim H. S. Kim J. H. Yim H. Kim D. (2012). Changes in the levels of interleukins 6, 8, and 10, tumor necrosis factor alpha,and granulocytecolony stimulating factor in Korean burn patients:relation to bum size and postburn time. Ann. Lab. Med. 32 (5), 339–344. doi: 10.3343/alm.2012.32.5.339 22950069 PMC3427821

[B55] Lazar V. Garcia J. G. (1999). A single human myosin light chain kinase gene (MLCK; MYLK). Genomics 57 (2), 256–267. doi: 10.1006/geno.1999.5774 10198165

[B56] Liu A. Minasian R. A. Maniago E. Gillenwater T. J. Garner W. L. Yenikomshian H. A.. (2021). Venous thromboembolism chemoprophylaxis in burn patients: A literature review and single-institution experience. J. Burn. Care Res. 42 (1), 18–22. doi: 10.1093/jbcr/iraa143 32842151

[B57] Liu R. T. Rowan-Nash A. D. Sheehan A. E. Walsh R. F. L. Sanzari C. M. Korry B. J.. (2020). Reductions in anti-inflammatory gut bacteria are associated with depression in a sample of young adults. Brain Behav. Immun. 88, 308–324. doi: 10.1016/j.bbi.2020.03.026 32229219 PMC7415740

[B58] Liu Y. L. Wang F. J. Chen C. L. Wang P. (2008). Increased intestinal permeability in severely burnt rats: regulatory mechanism of rho kinase. Acta Academ. Med. Milit. Tert. 30 (9), 817–819. doi: 10.3321/j.issn:1000-5404.2008.09.012

[B59] Li G. Wang S. Fan Z. (2021). Oxidative stress in intestinal ischemia-reperfusion. Front. Med. (Lausanne). 8, 750731. doi: 10.3389/fmed.2021.750731 35096858 PMC8795364

[B60] Maes M. Galecki P. Chang Y. S. Berk M. (2011). A review on the oxidative and nitrosative stress (O&NS) pathways in major depression and their possible contribution to the (neuro)degenerative processes in that illness. Prog. Neuropsychopharmacol. Biol. Psychiatry 35 (3), 676–692. doi: 10.1016/j.pnpbp.2010.05.004 20471444

[B61] Mawdsley J. E. Rampton D. S. (2005). Psychological stress in IBD: new insights into pathogenic and therapeutic implications. Gut 54 (10), 1481–1491. doi: 10.1136/gut.2005.064261 16162953 PMC1774724

[B62] Mi L. Zhang N. Wan J. Cheng M. Liao J. Zheng X. (2021). Remote ischemic post−conditioning alleviates ischemia/reperfusion−induced intestinal injury *via* the ERK signaling pathway−mediated RAGE/HMGB axis. Mol. Med. Rep. 24 (5), 773. doi: 10.3892/mmr.2021.12413 34490475 PMC8441982

[B63] Morarasu S. Morarasu B. C. Ghetu N. Dimofte M. G. Iliescu R. Pieptu D. (2021). Experimental models for controlled burn injuries in rats: A systematic analysis of original methods and burn devices. J. Burn. Care Res. 43 (5), 1055–1065. doi: 10.1093/jbcr/irab234 34888684

[B64] Moses T. Wagner L. Fleming S. D. (2009). TLR4-mediated cox-2 expression increases intestinal ischemia/reperfusion-induced damage. J. Leukoc. Biol. 86 (4), 971–980. doi: 10.1189/jlb.0708396 19564573 PMC2752016

[B65] Nadatani Y. Watanabe T. Shimada S. Otani K. Tanigawa T. Fujiwara Y.. (2018). Microbiome and intestinal ischemia/reperfusion injury. J. Clin. Biochem. Nutr. 63 (1), 26–32. doi: 10.3164/jcbn.17-137 30087540 PMC6064812

[B66] Peppas S. Pansieri C. Piovani D. Danese S. Peyrin-Biroulet L. Tsantes A. G.. (2021). The brain-gut axis: Psychological functioning and inflammatory bowel diseases. J. Clin. Med. 10 (3), 377. doi: 10.3390/jcm10030377 33498197 PMC7863941

[B67] Piao L. Zhao G. Zhu E. Inoue A. Shibata R. Lei Y.. (2017). Chronic psychological stress accelerates vascular senescence and impairs ischemia-induced neovascularization: The role of dipeptidyl peptidase-4/Glucagon-Like peptide-1-Adiponectin axis. J. Am. Heart Assoc. 6 (10), e006421. doi: 10.1161/JAHA.117.006421 28963101 PMC5721852

[B68] Rizzo J. A. Rowan M. P. Driscoll I. R. Chung K. K. Friedman B. C.. (2016). Vitamin c in burn resuscitation. Crit. Care Clin. 32 (4), 539–546. doi: 10.1016/j.ccc.2016.06.003 27600125

[B69] Shen L. Weber C. R. Raleigh D. R. Yu D. Turner J. R.. (2011). Tight junction pore and leak pathways: a dynamic duo. Annu. Rev. Physiol. 73, 283–309. doi: 10.1146/annurev-physiol-012110-142150 20936941 PMC4655434

[B70] Sluzewska A. Rybakowski J. Bosmans E. Sobieska M. Berghmans R. Maes M.. (1996). Indicators of immune activation in major depression. Psychiatry Res. 64 (3), 161–167. doi: 10.1016/S0165-1781(96)02783-7 8944394

[B71] Smolle C. Cambiaso-Daniel J. Forbes A. A. Wurzer P. Hundeshagen G. Branski L.K.. (2017). Recent trends in burn epidemiology worldwide: a systematic review. Burns 43 (2), 249–257. doi: 10.1016/j.burns.2016.08.013 27600982 PMC5616188

[B72] Suzuki K. Nakamura K. Shimizu Y. Yokoi Ohira S. Hagiwara M.. (2021). Decrease of α-defensin impairs intestinal metabolite homeostasis *via* dysbiosis in mouse chronic social defeat stress model. Sci. Rep. 11 (1), 9915. doi: 10.1038/s41598-021-89308-y 33972646 PMC8110768

[B73] Takeo M. Lee W. Ito M. (2015). Wound healing and skin regeneration. Cold Spring Harb. Perspect. Med. 5 (1), a023267. doi: 10.1101/cshperspect.a023267 25561722 PMC4292081

[B74] Walf A. A. Frye C. A. (2007). The use of the elevated plus maze as an as-say of anxiety-related behavior in rodents. Nat. Protoc. 2 (2), 322–328. doi: 10.1038/nprot.2007.44 17406592 PMC3623971

[B75] Wang H. An X. Yu H. Zhang S. Tang B. Zhang X.. (2017). MiR-29b/TET1/ZEB2 signaling axis regulates metastatic properties and epithelial-mesenchymal transition in breast cancer cells. Oncotarget 8 (60), 102119–102133. doi: 10.18632/oncotarget.22183 29254230 PMC5731940

[B76] Wang G. Chen Z. Zhang F. Jing H. Xu W. Ning S.. (2014). Blockade of PKCβ protects against remote organ injury induced by intestinal ischemia and reperfusion *via* a p66shc-mediated mitochondrial apoptotic pathway. Apoptosis 19 (9), 1342–1353. doi: 10.1007/s10495-014-1008-x 24930012

[B77] Wang C. Li Q. Ren J. (2019). Microbiota-immune interaction in the pathogenesis of gut-derived infection. Front. Immunol. 10, 1873. doi: 10.3389/fimmu.2019.01873 31456801 PMC6698791

[B78] Wang H. Meng X. Piao L. Inoue A. Xu W. Yu C.. (2019). Cathepsin s deficiency mitigated chronic stress-related neointimal hyperplasia in mice. J. Am. Heart Assoc. 8 (14), e011994. doi: 10.1161/JAHA.119.011994 31296090 PMC6662117

[B79] Wei L. Li Y. Tang W. Sun Q. Chen L. Wang X.. (2019). Chronic unpredictable mild stress in rats induces colonic inflammation. Front. Physiol. 10, 1228. doi: 10.3389/fphys.2019.01228 31616319 PMC6764080

[B80] Wen S. H. Li Y. Li C. Xia Z. Q. Liu W. F. Zhang X.. (2012). Ischemic postconditioning during reperfusion attenuates intestinal injury and mucosal cell apoptosis by inhibiting JAK/STAT signaling activation. Shock 38 (4), 411–419. doi: 10.1097/SHK.0b013e3182662266 22777122

[B81] Willner P. Towell A. Sampson D. Sophokleous S. Muscat R. (1987). Reduction of sucrose preference by chronic unpredictable mild stress, and its restoration by a tricyclic antidepressant. Psychopharmacol. (Berl). 93, 358–364. doi: 10.1007/BF00187257 3124165

[B82] Wong Y. L. Lautenschläger I. Hummitzsch L. Zitta K. Cossais F. Wedel T.. (2021). Effects of different ischemic preconditioning strategies on physiological and cellular mechanisms of intestinal ischemia/reperfusion injury: Implication from an isolated perfused rat small intestine model. PloS One 16 (9), e0256957. doi: 10.1371/journal.pone.0256957 34478453 PMC8415612

[B83] Xue Y. He J. T. Zhang K. K. Chen L. J. Wang Q. Xie X. L.. (2019). Methamphetamine reduces expressions of tight junction proteins, rearranges f-actin cytoskeleton and increases the blood brain barrier permeability *via* the RhoA/ROCK-dependent pathway. Biochem. Biophys. Res. Commun. 509 (2), 395–401. doi: 10.1016/j.bbrc.2018.12.144 30594393

[B84] Xu D. Gao J. Gillilland M. Wu X. Song L. Kao J.. (2014). Rifaximin alters intestinal bacteria and prevents stress-induced gut inflammation and visceral hyperalgesia in rats. Gastroenterology 146 (2), 484–496. doi: 10.1053/j.gastro.2013.10.026 24161699 PMC3939606

[B85] Xu H. Liu L. Tian Y. Wang J. Li J. Zheng J.. (2019). A disinhibitory microcircuit mediates conditioned social fear in the prefrontal cortex. Neuron 102 (3), 668–682.e5. doi: 10.1016/j.neuron.2019.02.026 30898376

[B86] Yan Y. Wang Y. L. Su Z. Zhang Y. Guo S. X. Liu A. J.. (2014). Effect of oxytocin on the behavior-al activity in the behavioral despair depression rat model. Neuropeptides 48 (2), 83–89. doi: 10.1016/j.npep.2014.01.001 24444823

[B87] Ye D. Ma T. Y. (2008). Cellular and molecular mechanisms that mediate basal and tumor necrosis factor-α induced regulation of myosin light chain kinase gene activity. J. Cell Mol. Med. 12 (4), 1331–1346. doi: 10.1111/j.1582-4934.2008.00302.x 18363837 PMC3865676

[B88] Yu E. P. Bennett M. R. (2016). The role of mitochondrial DNA damage in the development of atherosclerosis. Free Radic. Biol. Med. 100, 223–230. doi: 10.1016/j.freeradbiomed.2016.06.011 27320189

[B89] Yue X. Piao L. Wang H. Huang Z. Meng X. Sasaki T.. (2022). Cathepsin K deficiency prevented kidney damage and dysfunction in response to 5/6 nephrectomy injury in mice with or without chronic stress. Hypertension 79 (8), 1713–1723. doi: 10.1161/HYPERTENSIONAHA.122.19137 35726642 PMC9278705

[B90] Zahs A. Bird M. D. Ramirez L. Turner J. R. Choudhry M. A. Kovacs E. J.. (2012). Inhibition of long myosin light-chain kinase activation alleviates intestinal damage after binge ethanol exposure and burn injury. Am. J. Physiol. Gastrointest. Liver. Physiol. 303 (6), G705–G712. doi: 10.1152/ajpgi.00157.2012 22790598 PMC3468533

[B91] Zhang D. Zhu C. Fang Z. Zhang H. Yang J. Tao K.. (2020). Remodeling gut microbiota by clostridium butyricum (C.butyricum) attenuates intestinal injury in burned mice. Burns 46 (6), 1373–1380. doi: 10.1016/j.burns.2020.01.007 32014349

[B92] Zheng P. Y. Feng B. S. Oluwole C. Struiksma S. Chen X. Li P.. (2009). Psychological stress induces eosinophils to produce corticotrophin releasing hormone in the intestine. Gut 58 (11), 1473–1479. doi: 10.1136/gut.2009.181701 19651632

[B93] Zhi L. Hu X. Xu J. Yu C. Shao H. Pan X.. (2015). The characteristics and correlation between the ischemia-reperfusion and changes of redox status in the early stage of severe burns. Am. J. Emerg. Med. 33 (3), 338–343. doi: 10.1016/j.ajem.2014.11.026 25552460

[B94] Zhu Q. He G. Wang J. Wang Y. Chen W. Guo T.. (2017). Down-regulation of toll-like receptor 4 alleviates intestinal ischemia reperfusion injury and acute lung injury in mice. Oncotarget 8 (8), 13678–13689. doi: 10.18632/oncotarget.14624 28099145 PMC5355129

[B95] Zhu E. Hu L. Wu H. Piao L. Zhao G. Inoue A.. (2017). Dipeptidyl peptidase-4 regulates hematopoietic stem cell activation in response to chronic stress. J. Am. Heart Assoc. 6 (7), e006394. doi: 10.1161/JAHA.117.006394 28710180 PMC5586325

[B96] Zhu Q. Qian X. Wang S. Yin T. Yang J. Xue Q.. (2006). A comparison of elderly and adult multiple organ dysfunction syndrome in the rat model. Exp. Gerontol. 41 (8), 771–777. doi: 10.1016/j.exger.2006.05.010 16797904

